# First report of *Crenosoma vulpis* in Africa and *Eucoleus aerophilus* in Algeria

**DOI:** 10.1016/j.ijppaw.2023.03.003

**Published:** 2023-03-08

**Authors:** Noureddine Mechouk, Georgiana Deak, Angela Monica Ionică, Corina Gina Toma, Zihad Bouslama, Andrei Daniel Mihalca

**Affiliations:** a Department of Parasitology and Parasitic Diseases, University of Agricultural Sciences and Veterinary Medicine of Cluj-Napoca, Calea Mănăștur 3-5, 400372, Cluj Napoca, Romania; b Laboratory of Ecology of Terrestrial and Aquatics Systems (EcoSTAq), Department of Biology, Faculty of Science, Badji Mokhtar University, BP 12, 23200, Annaba, Algeria; c Molecular Biology and Veterinary Parasitology Unit (CDS 9), “Regele Mihai I Al României” Life Science Institute, University of Agricultural Sciences and Veterinary Medicine of Cluj-Napoca, Calea Mănăştur 3-5, 400372, Cluj Napoca, Romania; d Molecular Diagnosis Laboratory, Clinical Hospital of Infectious Diseases of Cluj-Napoca, 23 Iuliu Moldovan, 400348, Cluj-Napoca, Romania; e Department of Veterinary Pathology, University of Agricultural Sciences and Veterinary Medicine of Cluj-Napoca, Calea Mănăștur 3-5, 400372, Cluj Napoca, Romania; f National Environmental Research Center, Sidi Amar Campus, BP N° 2024, 23005, Annaba, Algeria

**Keywords:** *Crenosoma vulpis*, Carnivores, *Eucoleus aerophilus*, Red fox, Algeria, Africa

## Abstract

**Introduction:**

*Crenosoma vulpis* and *Eucoleus aerophilus* are widely distributed lungworms infecting carnivores, mainly red foxes, and are localized in the trachea, bronchi and bronchioles. There are no reports from Africa regarding the presence of *C. vulpis*. The aim of the present study was to report a co-infection with *C. vulpis* and *E. aerophilus* in a red fox from Algeria.

**Materials and methods:**

In January 2022, a road-killed male juvenile red fox (*Vulpes vulpes*) was collected from Bouhadjar-Tarf locality and was submitted for a complete parasitological necropsy. Detected nematodes were collected and preserved in ethanol for morphological and molecular identification. Tissue samples were also collected and analyzed by histopathological methods.

**Results:**

Collected nematodes were identified as a male *C. vulpis* and several *Eucoleus aerophilus*. The histological techniques of the lung tissue did not reveal the presence of any larvae, or lung inflammation.

**Conclusion:**

To the best of our knowledge, this is the first report of *C. vulpis* infecting a carnivore in this continent, highlighting the importance of further studies to update the geographical distribution of *C. vulpis*. *E. aerophilus* was first reported in Algeria. Red foxes are important spreaders of parasitic diseases. Further studies are required for a better understanding of its epidemiology across North Africa and other areas overlapping the range of the red fox.

## Highlights

• First report of *Crenosoma vulpis* in Africa.
• First report of *Eucoleus aerophilus* in Africa.
• New geographical distribution of *Crenosoma vulpis* and *Eucoleus aerphilus*.

## Graphical abstract

**Figure:**
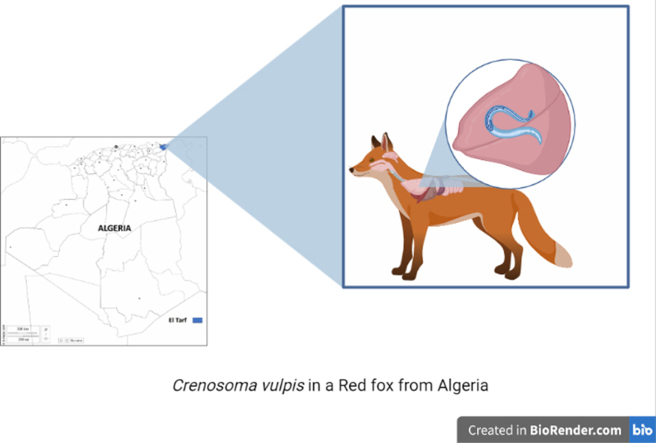

## 1 Introduction

Algeria is the biggest country in Africa with diverse biogeographic regions. With such characteristics, Algeria hosts 21 carnivorous mammal species, including red fox (*Vulpes vulpes* L. 1758), which is distributed mainly in the northern part of the country (Ahmim, 2019). These mammals act as major predators and their diet (Kidawa and Kowalczyk, 2011), exposes them to a large range of parasites (Veronesi et al., 2023), including metastrongyloid lungworms like *Angiostrongylus vasorum*, *Crenosoma vulpis* (Strongylida: Crenosomatidae) and *Eucoleus aerophilus* (Anderson, 2000). A recent study done in a geographically close country (Tunisia) evaluated the diet of red foxes by analysing the faeces and showed that the main food source were invertebrates and rodents (Karssene et al., 2019).

The fox lungworm, *C. vulpis*, is a metastrogyloid nematode with an indirect life cycle which was so far reported in North and South America, Europe, and Central Asia. Vertebrate hosts get infected by oral ingestion of different gastropod species containing third-stage larvae (L3), which act as intermediate hosts (Conboy, 2004; Penagos-Tabares et al., 2019; Deak et al., 2020; Safarov et al., 2022). There are no studies or reports of its presence in Africa. This nematode species is localized in the trachea, bronchi and bronchioles of a wide range of carnivores (Reilly et al., 2000) (Goble and cook. 1942; Zeh et al., 1977). Although wild canids are not clinically affected by the infection with *C. vulpis*, respiratory distress has been observed in dogs (Stockdale and Hulland, 1970; Cobb and Fisher, 1992; Hoff, 1993; Reilly et al., 2000).

*Eucoleus aerophilus* is a thin capillariid nematode infecting the trachea and bronchi of red foxes (Deak et al., 2000) and other carnivore and insectivorous animal species (Anderson, 2000) with wide geographical distribution (Traversa et al., 2010). In contrast to *C. vulpis*, its life cycle is not completely known yet, but it seems to be transmitted either by direct faecal-oral route or by ingesting paratenic hosts (oligochaetes) (Anderson, 2000; Deplazes et al., 2016). Although very different regarding their biology, the two lungworms seem to be common parasites of red foxes and are often co-distributed (Deak et al., 2020).The aims of the present paper were to provide new data regarding the geographical distribution of *C. vulpis* and *E. aerophilus*.

## 2 Materials and methods

In January 2022, a road-killed male juvenile red fox (*Vulpes vulpes*) was collected from the Bouhadjar EL-Tarf locality (36° 30′ 11″ N, 8° 6′ 19″ E) and transferred to the National Environmental Research Center. The carcass was stored in a labelled plastic bag at −20 °C until processing. A full parasitological necropsy was performed, and details regarding the age of the animal and sexual maturity were recorded (Klevezalʹ and Kleĭnenberg, 1967; Roulichova and Andera 2007). The entire respiratory tract was removed, and the trachea, bronchi and bronchioles were longitudinally opened and scrutinized for parasites under a stereomicroscope. Detected helminths were collected in a 2 ml labelled tube with absolute ethanol and preserved until identification. In addition, 2 cm of lung tissue samples were collected in 10% formalin, embedded in paraffin wax, cut into 2–3 μm thick sections, and stained with hematoxylin and eosin (H&E) for histopathological examination. The nematodes were transferred to the Department of Parasitology and Parasitic Diseases of the University of Agriculture and Veterinary Medicine of Cluj-Napoca for morphological and molecular identification. The collected nematodes were temporarily mounted in mineral oil and morphologically identified based on specific descriptions (Jančev and Genov, 1988; Traversa et al., 2011). Genomic DNA was extracted from one specimen of each type of nematode using a commercial kit (Isolate II Genomic DNA Kit, meridian Bioscience, London, UK) according to the producer's instructions. For each of the nematodes, a PCR amplification of a fragment of the *cytochrome c oxidase* subunit 1 gene (*cox*1) was performed, using the universal primers LCO1490/HCO2198 for *Crenosoma* sp., and Cox1NEMF [S0823]/Cox1NEMR [S0824] for *E. aerophilus*, according to literature (Folmer et al., 1994; Di Cesare et al., 2012). The PCR products were sequenced using an external service (performed by Macrogen Europe, Amsterdam, The Netherlands) and compared to others available in GenBank using Basic Local Alignment Search Tool (BLAST) analysis.

The distribution map was elaborated using ArcMap 10.6.1. The graphical abstract was made using Biorender.

## 3 Results

**Fig. 1:**
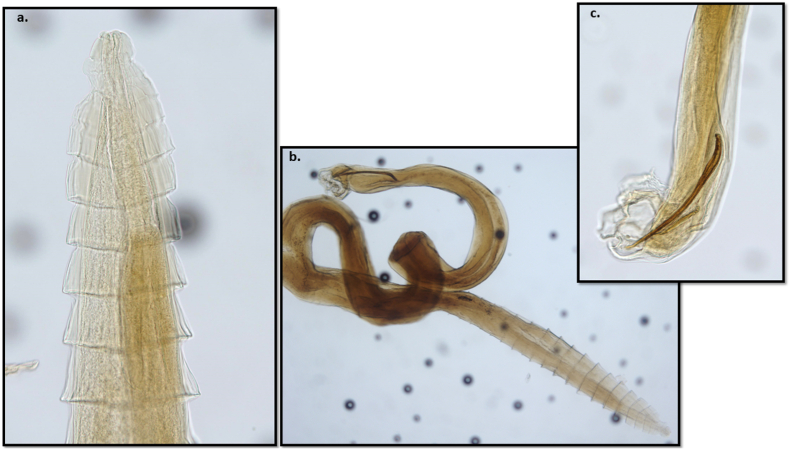
**Morphological characteristics of *Crenosoma vulpis* male**. a) The anterior part of the nematode showing specific cuticular folds; b) the general appearance of the male nematode; c) posterior end of the worm showing the copulatory bursa and the spicules.

**Fig. 2:**
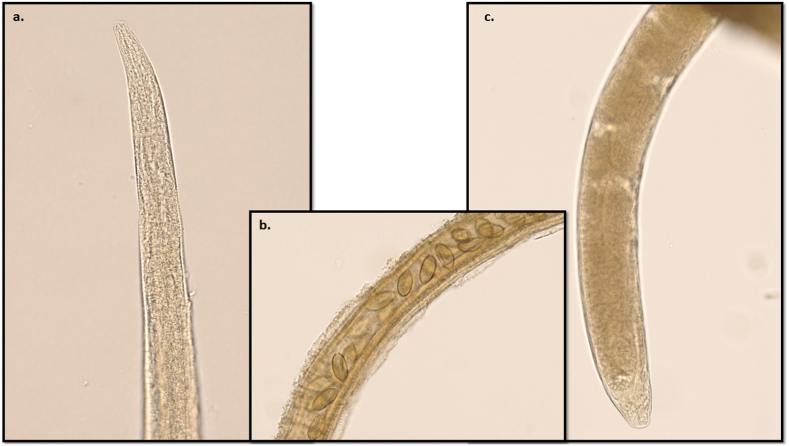
**Adult female *Eucoleus aerophilus***. a) the anterior extremity; b) note the uterus of the specimen containing lemon-shaped eggs; c) the posterior extremity.

Overall, seven nematodes were detected in the trachea and bronchi of the fox and were morphologically identified as a single male *Crenosoma vulpis* and 4 females and 2 male *Eucoleus aerophilus* (Fig. 1). The histopathological examination did not reveal the presence of any parasitic form in the lung tissues examined. Moreover, no inflammation or other pathological modifications were observed (see Fig. 2).

The BLAST analysis showed 99.76–97.3% nucleotide identity to *Crenosoma vulpis* isolates (Accession no. **ON965049-ON965052**) for *Crenosoma* sp., while the second sequence had a 100% homology to other *E. aerophilus* isolates (Accession no. **MW709563**- **MW709567**).

## 4 Discussion

**Fig. 3:**
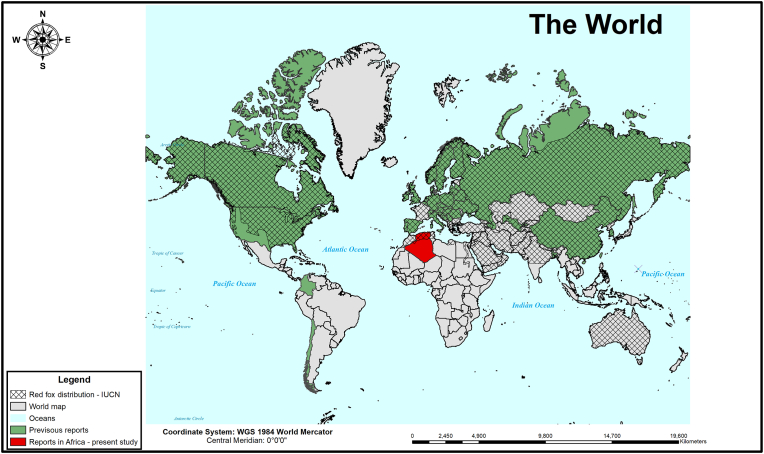
**Map showing the global distribution of *Crenosoma vulpis* reported in vertebrate and invertebrate hosts**. The positive countries for *C. vulpis* were marked based on the following references: (Rankin and AuthorAnonymous, 1946; Borgsteede, 1984; Rausch et al., 1990; Alvarez et al., 1991; Peterson et al., 1993; Hoff 1993; Bihr and Conboy, 1999; Reilly et al., 2000; Conboy 2004; Ridyard 2005; Davidson et al., 2006; Rinaldi et al., 2007; Mörner and Christensson, 2007; Nelson et al., 2007; Subbotin et al., 2008; Barutzki and Schaper, 2009; Popiołek et al., 2009; Taubert et al., 2009; Bagrade et al., 2009; Conboy, 2009; Shahba, 2010; Fataliyev, 2011; Simin et al., 2012; Bružinskaitė-Schmidhalter et al., 2012; Karkamo et al., 2012; Vergles Rataj et al., 2013; Oyarzún-Cadagán, 2013; Choi et al., 2014; Yousuf et al., 2014; Tolnai et al., 2015; Lempereur et al., 2016; Hodžić et al., 2016; Bosančić, 2016; Hajnalová et al., 2017; Varodi et al., 2017; Čabanová et al., 2018; Figueiredo et al., 2018; Rice 2018; Gavrilović et al., 2019; Каменов and Шинкаренко, 2019; Penagos-Tabares et al., 2019; Deak et al., 2020; Fuehrer et al., 2020; Gillis-Germitsch et al., 2020; Pohly et al., 2022; Chen et al., 2022; Safarov et al., 2022). The locations of the red fox (*Vulpes vulpes*) world distribution were extracted according to IUCN (accessed on January 2023). (For interpretation of the references to colour in this figure legend, the reader is referred to the Web version of this article.)

This is the first evidence of the presence of *C. vulpis* in Africa. This lungworm species was previously reported in Europe, in North and South America and in Asia (Fig. 3) (Bihr and Conboy, 1999; Conboy, 2004; Penagos-Tabares et al., 2019; Stunžėnas and Binkienė, 2021; Pohly et al., 2022; Safarov et al., 2022). Interestingly, the reports of this nematode coincide with the known distribution of the red fox (*Vulpes vulpes*) (IUCN) and we could hypothesize that red foxes are the most important host species responsible for the spreading of *C. vulpis* and *E. aerophilus* to domestic habitat as well as to new geographical areas together with their expansion. Infection in red foxes is not responsible for a clinical picture, but domestic dogs can manifest chronic cough (Cobb and Fisher, 1992). The lack of reports of *C. vulpis* presence in other areas where red foxes are introduced (e.g. Australia) can be related to the lack of studies on this topic. Although a wide variety of gastropods can act as intermediate hosts for *C. vulpis*, climatic factors can influence larval development in non-vertebrate hosts and can serve as a reason for the absence of reports in some geographical areas. In the area from where this fox was collected, many gastropod species are present in a large number (Larbaa and Soltani, 2013). Of course, there are other reasons for the lack of reports, at least in domestic dogs, like the use of a less sensitive diagnostic method. Diagnosis of canine crenosomiasis is based on the concentration and morphological identification of L1 in the faeces using larvoscopic methods like the Baermann technique (Deplazes et al., 2016).

Following the same pattern, *E. aerophilus* shows a high prevalence in Europe in wolves (Estévez-Sánchez et al., 2022). Commonly, red foxes are often co-infected with the two lungworms, despite the differences in their biology. In the case of *E. aerophilus,* which also has a zoonotic character, the nematode has a direct and indirect life cycle. The infection occurs through the ingestion of eggs or paratenic hosts such as lumbricids (Deak et al., 2020). One severe case of human eucoleosis was reported in Morocco, another country from Northern Africa (Coudert et al., 1972).

Natural infection of red foxes with these lungworms seems to be benign (Goble and Cook, 1942; Zeh et al., 1977; Lalošević et al., 2013). However, dogs and cats can occasionally develop clinical manifestations represented by chronic respiratory disease (Stockdale and Hulland, 1970; Cobb and Fisher, 1992; Hoff, 1993; Reilly et al., 2000; Traversa et al., 2009). In Algeria, a considerable population of stray and free-roaming dogs occurs, and such animals play an important role in the epidemiology of diseases with public health impact, such as rabies (Abdi et al., 2006), echinococcosis (Mehrabani et al., 1999) or leishmaniasis (Müller et al., 2022).

## 5 Conclusion

To the best of our knowledge, this is the first report of the occurrence of *Crenosoma vulpis* in Africa and *Eucoleus aerophilus* in Algeria, Northern Africa, extending their known distribution range to a new continent. The authors would like to highlight the role of red foxes in the spreading of pathogens and advocate for more parasitological studies in the future for controlling diseases.

## Funding

The work of AMI was supported by a grant agency of the Ministry of Research, Innovation, and Digitization, CNCS-UEFISCDI, project number PN-III-P1-1.1-TE-2021-0519, contract TE49/2022, within PNCDI III.

## Author contributions

NM & GD performed the necropsies GD morphologically identified the nematodes, performed the PCRs and revised the manuscript. AMI performed the PCR and revised the manuscript. CGT performed the histopathological examination and revised the manuscript. ZB and ADM coordinated the study, revised and approved the final version of the manuscript. All authors read and approved the final manuscript.

## Declaration of competing interest

The authors have nothing to declare.
